# Negative pressure wound therapy in the treatment of diabetic foot ulcers may be mediated through differential gene expression

**DOI:** 10.1007/s00592-018-1223-y

**Published:** 2018-09-17

**Authors:** S. Borys, A. H. Ludwig-Slomczynska, M. Seweryn, J. Hohendorff, T. Koblik, J. Machlowska, B. Kiec-Wilk, P. Wolkow, Maciej T. Malecki

**Affiliations:** 1 Department of Metabolic Diseases, Jagiellonian University Medical College, 15 Kopernika Street, 31-501 Kraków, Poland; 2 University Hospital, Kraków, Poland; 3 Center for Medical Genomics OMICRON, Jagiellonian University Medical College, Kraków, Poland

**Keywords:** Diabetic foot syndrome, Wound healing, NPWT, Gene expression

## Abstract

**Aims:**

Negative pressure wound therapy (NPWT) has been successfully used as a treatment for diabetic foot ulceration (DFU). Its mechanism of action on the molecular level, however, is not fully understood. We assessed the effect of NPWT on gene expression in patients with type 2 diabetes (T2DM) and DFU.

**Methods:**

We included two cohorts of patients—individuals treated with either NPWT or standard therapy. The assignment to NWPT was non-randomized and based on wound characteristics. Differential gene expression profiling was performed using Illumina gene expression arrays and R Bioconductor pipelines based on the ‘limma’ package.

**Results:**

The final cohort encompassed 21 patients treated with NPWT and 8 with standard therapy. The groups were similar in terms of age (69.0 versus 67.5 years) and duration of T2DM (14.5 versus 14.4 years). We identified four genes differentially expressed between the two study arms post-treatment, but not pre-treatment: GFRA2 (GDNF family receptor alpha-2), C1QBP (complement C1q binding protein), RAB35 (member of RAS oncogene family) and SYNJ1 (synaptic inositol 1,4,5-trisphosphate 5-phosphatase 1). Interestingly, all four genes seemed to be functionally involved in wound healing by influencing re-epithelialization and angiogenesis. Subsequently, we utilized co-expression analysis in publicly available RNA-seq data to reveal the molecular functions of GFRA2 and C1QBP, which appeared to be through direct protein–protein interactions.

**Conclusions:**

We found initial evidence that the NPWT effect on DFUs may be mediated through differential gene expression. A discovery of the specific molecular mechanisms of NPWT is potentially valuable for its clinical application and development of new therapies.

## Introduction

Diabetic foot syndrome (DFS) exhibits a complex underlying pathophysiology. It is characterized as an infection, ulceration or destruction of deep tissues of the foot associated with neuropathy and/or peripheral arterial disease [1]. DFS, commonly occurring with ulcerations, is associated with a high rate of relapse, amputation and mortality [2–4]. Despite new therapeutic advances, many patients still develop various forms of DFS at different stages.

Standard therapy of diabetic foot ulceration (DFU) includes glycemic control, offloading, revascularization, systemic antibiotics, surgical debridement, and different topical applications [5]. Negative pressure wound therapy (NPWT) has attracted attention as an approach in the treatment for DFU. NPWT utilizes a vacuum dressing to accelerate wound healing, a process consisting of classical stages—hemostasis, inflammation, proliferation, and maturation. Improvement of blood flow, induction of wound contraction, promotion of granulation and angiogenesis, increased wound fluid removal with a decrease in local edema, and bacterial colonization reduction have all been postulated as benefits of NPWT [6]. The specific molecular mechanisms of NPWT, potentially valuable for the application and development of new therapies, remain poorly understood. One hypothesis has proposed that NPWT acts through alteration in the gene expression profile. This has been supported in animal models and preliminarily in humans [7–13], however, has never been systematically examined.

In this study, we assessed the effect of NPWT on the gene expression profile in the wound bed of patients with type 2 diabetes presenting with DFU.

## Subjects and methods

### Study population

Patients were recruited from an outpatient clinic specializing in diabetic foot care. We included 36 consecutive patients with type 2 diabetes and DFU. They were assigned to either the standard therapy alone or combined with NPWT for 8 ± 1 days. The assignment to NWPT was non-random and based on wound characteristics. The inclusion criteria comprised of (a) a clinical diagnosis of type 2 diabetes and (b) the presence of no more than three neuropathic, clinically noninfected foot wounds. Exclusion criteria included (a) clinically significant ischemia defined by the lack of pulses of both main pedal arteries and/or an ankle–brachial index less than 0.9, (b) symptoms of infection, (c) bilateral ulcerations, (d) active osteomyelitis, and (e) active Charcot foot.

We assigned patients with type 2 diabetes, presenting with at least one ulceration with a size greater than 1 cm^2^ to NPWT, while those with ulcerations less than 1 cm^2^, to the comparator group. However, in case of technical difficulties (presence of very large ulcerations greater than 1 cm^2^, unfavorable localizations) or lack of consent to NPWT, patients were allocated to the comparator group.

During the initial visit, each study participant was assigned to one of the arms and an initial (pre-treatment) tissue sample from the wound bed; a blood specimen for basic biochemical measurements was also collected. Change of the NPWT dressing was performed 3–5 days later. Finally, at day 8 ± 1, the second (post-treatment) wound tissue samples were taken. In the control arm, the samples were taken on the same days (0 and 8 ± 1). Clinical data were compiled from available medical records.

The study protocol was approved by the Jagiellonian University Bioethical Committee and was in accordance with the Declaration of Helsinki. Patients’ written informed consent was obtained prior to inclusion.

### Patients’ baseline characteristics analysis

Statistical analysis was performed using Statistica Software v. 12.0 (StatSoft, Tulsa, OK, USA). A *p* value of < 0.05 was considered significant. Parametric *t* tests, nonparametric *U* tests and Chi-square tests were performed to describe baseline clinical characteristic of the study groups. Wound area was measured using MOWA Mobile Wound Analyzer (Healthpath, Italy) application.

### Gene expression quantification

After collection, tissue samples were placed in an RNAlater solution (Ambion, Foster City, CA, USA). Total RNA was extracted using the Maxwell instrument (Promega, Madison, WI, USA). RNA quality was determined with Tape Station (Agilent, Santa Clara, CA, USA) and its quantity with Quantus (Promega, Madison, WI, USA). Reverse transcription (first- and second-strand synthesis), followed by in vitro production of biotin–aRNA was performed using Target NanoAmp Labelling Kit (Epicenter, Madison, WI, USA). After purification, 750 ng of aRNA was hybridized to an Illumina Human HT-12v4 chip (Illumina, San Diego, CA, USA) according to the manufacturer’s protocol. Arrays were scanned on the HiScan scanner (Illumina, San Diego, CA, USA).

### Differential expression analysis

For normalization, filtration, as well as testing of differential expression, we applied the standard approach based on ‘beadarray’, ‘lumi’ and ‘limma’ packages in R. In short, data were uploaded in the .IDAT format, low-quality probes and samples were removed, and background correction and log2 transformation with quantile normalization were applied. We analyzed the following linear modes:

1. expression ~ 1 + treatment_status + treatment_status:study_arm;
2. expression ~ 1 + study_arm + study_arm:treatment_status.

The first mode was used to estimate the effect of treatment (regardless of study arm) and the post-treatment contrast between the study arms, whereas the second model was used to estimate the arm-specific effect and the contrast between pre- and post-treatment expressions in each study arm separately. The Benjamini–Hochberg correction was applied. Co-expression analyses in GTEx data were done using two R packages: ‘mglR’ and ‘psych’. We used the Pearson product-moment correlation coefficient and the Benjamini–Hochberg correction.

## Results

In the initial cohort, there were 25 patients treated with NPWT (for a total 50 samples) and 11 patients were treated with conventional therapy (22 samples). Due to a number of poor-quality samples identified, 14 of them were removed—6 control and 8 NPWT (in the paired sample design, we removed the post- and pre-treatment samples if one of them was of poor quality). The final analysis comprised of 21 patients treated with NPWT and 8 individuals treated with standard therapy. The groups were not different in terms of time between the initial and final visits, as well as basic clinical characteristics (Table 1).

**Table 1:** Characteristics of the study groups

	NPWT	Standard therapy	*p* value
*n*	21	8	NA
Sex, *n* male/female, male%	17/4, 80.95%	6/2, 75.00%	0.7236
Age at examination, years^a^	69.0 ± 8.3	67.5 ± 4.3	0.6235
Diabetes duration, years^a^	14.5 ± 7.0	14.4 ± 5.7	0.9789
BMI, kg/m^2 a^	27.7 ± 4.6	31.8 ± 6.1	0.0782
Insulin therapy, *n* Y/N, Y%	19/2, 90.5%	8/0, 100.0%	0.3657
Total daily insulin dose, units^1^	45.1 ± 22.8	60.8 ± 17.7	0.1416
Total daily insulin dose, units/kg body weight^a^	0.52 ± 0.26	0.69 ± 0.22	0.1778
HbA1c, mmol/mol, (%)^a^	51.8 ± 14.7 (6.89 ± 1.34)	64.5 ± 20.5 (8.05 ± 1.86)	0.0725
eGFR, ml/min/1.73 m^2^ (CKD EPI)^a^	75.9 ± 20.0	70.0 ± 18.5	0.4780
Smoking, *n* (never/former/current)	5/11/2	4/3/1	0.5034
Wound area, cm^2 a^	18.8 ± 17.3	3.7 ± 6.1	0.0247

*n* Number of patients

^a^Data shown as mean ± SD

**Fig. 1:**
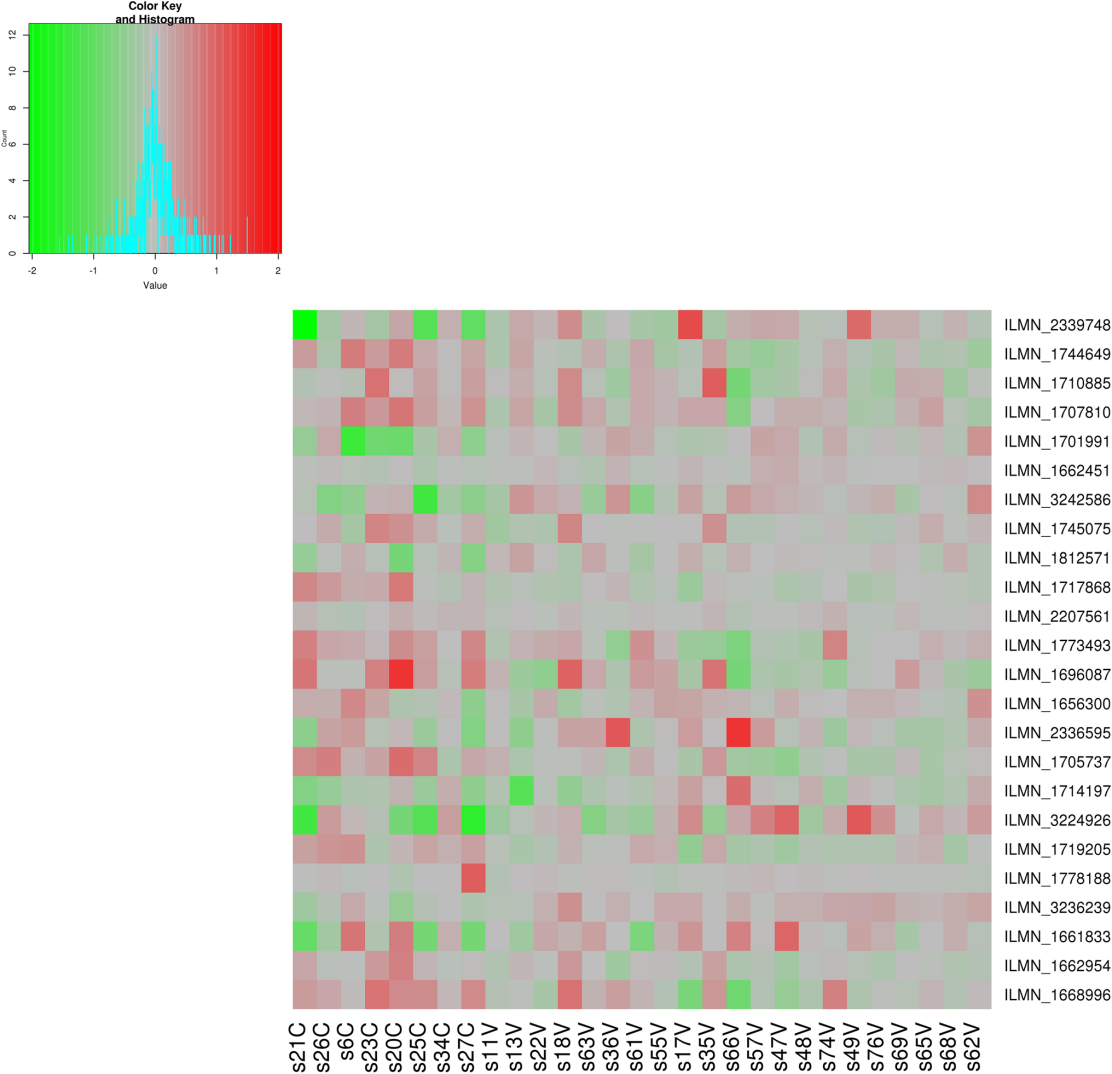
This heatmap illustrates 24 probes with the largest post-treatment difference (FDR < 0.1) in gene expression between the groups. The markers are ranked according to their statistical significance. Columns 1–8 correspond to patients with type 2 diabetes treated with standard therapy and the remaining ones to individuals exposed to NPWT. The color corresponds to the post- versus pre-treatment difference in normalized expression values for each patient–probe combination

After removal of all weak signal probes, 34,476 out of 48,107 probes remained for analysis. We identified four probes (ILMN_1656300, ILMN_1668996, ILMN_1812571 and ILMN_1701991) differentially expressed between the study arms (FDR ≤ 0.055) post-treatment, but not pre-treatment. These probes mapped to four mRNAs: GFRA2 (GDNF family receptor alpha-2), a member of the glial cell line-derived neurotrophic factor receptor family, C1QBP (complement component 1, q subcomponent-binding protein), RAB35 (member of RAS oncogene family) and SYNJ1 (synaptic inositol 1,4,5-trisphosphate 5-phosphatase 1). When compared to the control arm, expression of C1QBP was higher in the NPWT cohort (logFC = 0.62), whereas the remaining genes were downregulated (logFC = − 0.38, − 0.30 and − 0.50, respectively) (Table 2). Figure 1 shows a heatmap depicting 24 markers with the largest differential gene expression between the groups.

**Table 2:** Probes differentially expressed between study arms post-treatment

IlluminaID	logFC	AveExpr.	*p* value	FDR	Gene name
ILMN_1668996	0.62	7.46	1.48E−06	0.028	C1QBP
ILMN_1656300	− 0.38	6.11	1.63E−06	0.028	GFRA2
ILMN_1812571	− 0.3	6.82	4.15E−06	0.048	RAB35
ILMN_1701991	− 0.5	6.74	6.34E−06	0.055	SYNJ1

At the same time, we did not detect differentially expressed probes between the study arms pre-treatment, nor with treatment status in the entire cohort. Moreover, we found no differentially expressed probes in linear model 2.

Since two of the four differentially expressed genes, namely C1QBP and GFRA2, function through direct protein–protein interactions, we further evaluated the molecular context of differentially expressed genes by performing co-expression analysis using gene-level RPKMs (reads per kilobase per million mapped reads) from publicly available The Genotype Tissue Expression Project data (http://www.gtexportal.org).

We found that the GDNF mRNA is co-expressed with its receptors, albeit with GFRA2 mRNA stronger than with GFRA1: in ‘skin—sun exposed (lower leg)’ (*ρ* = 0.28 and 0.19; *p* = 3 × 10^−5^ and 1 × 10^−9^) and in ‘nerve tibial’ tissue (*ρ* = 0.77 and 0.49; *p* < 10^−12^ in both cases). On the other hand, NRTN (neurturin—a ligand for GFRA2) mRNA is weakly and negatively co-expressed (in both tissues) with GFRA1 and GFRA2 mRNAs.

We also found that C1QBP mRNA is robustly co-expressed with mRNAs of other components of the complement system (PRKCZ, C1QBP, C1QA, C1QB, C1QC, C1R, C1S, C3, C4A, C4B) both in ‘Liver’ (*ρ* = 0.43, 0.52, 0.52, 0.54, 0.23, 0.25, 0.38, 0.49 and 0.55; all *p* values < 3 × 10^−3^) and ‘Whole Blood’ tissue (*ρ* = 0.11, 0.32, 0.31, 0.30, 0.23, 0.23, 0.17, 0.28 and 0.26; all *p* values < 3.8 × 10^−2^).

## Discussion

For the first time, the effect of NPWT on gene expression profile was assessed in patients with type 2 diabetes with foot ulcerations on the gene expression profile using human gene expression arrays. Below we discuss the possible involvement of differentially expressed genes in re-epithelialization and angiogenesis of the proliferation stage of wound healing.

Loosening cell–cell adhesion promotes the migratory potential of the cells. This may occur by the removal of proteins responsible for maintaining this contact such as E-cadherins from the cell surface. RAB35, one of the key regulators of intracellular transport, was downregulated in our patients with type 2 diabetes after NPWT. Interestingly, lower expression of RAB35 mRNA (e.g. through excitation of miR-720) in HeLa cells increases vimentin expression, reduces cell–cell adhesion, and promotes cell migration [14]. Knockdown of RAB35 in vitro leads to epithelial–mesenchymal transition [15].

Our results show a relative downregulation of SYNJ1 in type 2 diabetes patients on NPWT. SYNJ1 dephosphorylates two phosphoinositides (PIP2 and PIP3) which help recruit clathrin coats to the plasma membrane [16]. Clathrin plays a role in endocytosis of membrane proteins (i.e. Cad-11) and thus enables cell migration [17]. Therefore, we suppose that downregulation of SYNJ1 might influence clathrin coating and cadherin endocytosis.

We also recorded downregulation of GFRA2 in NPWT patients. It has been described that the application of GDNF cytokine, a ligand for two receptors—GFRA1 and GFRA2—to the wound site accelerates its healing (a US patent, number WO2014152511 A1, 2014). Wound healing potential of this particle might be a result of the stabilization of epithelial barrier function and an increase in proliferative potential [18]. We hypothesize that the downregulation of GFRA2 might result in preferential binding of GDNF to GFRA1 (its higher affinity receptor) and promote cell survival. This is supported by the co-expression analysis in GTEx data, showing a consistently higher co-expression between GDNF and GFRA2 as compared to GDNF and GFRA1.

The only upregulated gene in NPWT group was C1QBP. High levels of C1QBP are found in highly migratory breast cancer cells and its downregulation inhibits wound healing and cell migration. In vivo results in SCID mice suggest that C1QBP is required for obtaining metastatic potential as injection of C1QBP knockdown cells into these animals failed to form tumors [19].

There are scarce data on the molecular aspects of NPWT. Some earlier papers reported findings from various experiments involving specific pathophysiological pathways selected based on their putative role in wound healing; none of them, however, systematically analyzed the effect of NWPT on gene expression. Recently, a small randomized trial in humans showed that NPWT increased expression of cellular fibronectin and transforming growth factor-β1 in DFS wounds [11], both of which, interestingly, influence cell migration and proliferation. Moreover, data from an animal model suggested that NPWT could influence the expression of genes involved in angiogenesis which is also a part of the proliferative stage of wound healing [9]. A very recent study, based on granulation tissue biopsies in patients with T2DM, showed that mRNA levels of several growth factors, such as VEGF and TGF-β1, were significantly increased, while the levels of matrix metalloproteinases (MMP-1 and MMP-9) and TNFα were significantly downregulated after NPWT [20]. This is in general line, including our paper, with NPWT acting on the molecular level towards more pro-angiogenic and anti-inflammatory conditions.

This study is limited by its non-random nature. However, as we assessed gene expression as an outcome rather than clinical end points, such as healing or amputation, the impact of wound size on the study results might be non-existing. Other shortcomings of this study include its limited number of patients, a meaningful proportion of samples that did not pass the quality control, and the borderline statistical significance of the reported findings. All required corrections for multiple comparisons were, however, done while RNAse enzyme abundance could have contributed to methodological difficulties in specimens from the wound beds. This study may be also perhaps criticized for lacking a replication of the microarray findings. However, such a validation would have required an independent set of biological samples, which was beyond the scope of this project.

## Conclusions

In summary, we found initial evidence that the effect of NPWT in DFS may be mediated through differential gene expression of proteins involved in the wound healing process. This finding requires further confirmation in subsequent studies.

## Abbreviations

CAD-11: Cadherin-11
C1QBP: Complement component 1, q subcomponent-binding protein
DFS: Diabetic foot syndrome
DFU: Diabetic foot ulceration
GDNF: Glial cell line-derived neurotrophic factor
GFRA1: GDNF family receptor alpha 1
GFRA2: GDNF family receptor alpha 2
IWGDF: The International Working Group on the Diabetic Foot
NPWT: Negative pressure wound therapy
PIP2: Phosphatidylinositol bisphosphate
PIP3: Phosphatidylinositol trisphosphate
RAB35: Ras-related protein 35
SCID: Severe combined immunodeficiency
